# How Many Children in a Family? Fertility Ideals and Gender Equality Norms in 26 Societies

**DOI:** 10.1007/s10680-026-09786-6

**Published:** 2026-09-08

**Authors:** Jonas Edlund, Ida Öun

**Affiliations:** Umeå University, Umeå, Sweden

**Keywords:** Fertility ideal, Gender equality, Norms, International social survey programme, ISSP, Gender revolution

## Abstract

We examine the relationship between gender equality and fertility ideals, focusing on the role of societal norms about men’s participation in care and household work, known as the second half of the gender revolution. We set out to capture how the prevalence of this revolution translates into fertility ideals on the societal level and among different social groups. Data come from the ISSP module 2012, covering 26 societies. The first objective is to examine the relationship between gender equality norms and fertility ideals across national contexts. The second objective is to compare how measures of gender equality focusing on the first half of the gender revolution, and the second half, respectively, are associated with fertility ideals. As previous research focusing on the first half of the gender revolution noted and/or predicted that actual or intended fertility tends to be comparatively lower among women, the highly educated, younger generations, and the non-religious, our third objective is to analyse the extent to which norms embracing the second half have the capacity to modify these relationships. The results show that norms about gender equality are positively related to fertility ideals and seem to influence patterns of fertility ideals among social groups. In societies with strong gender equality norms, women, the highly educated, and the young display comparatively higher fertility ideals. In societies with weak gender equality norms, these patterns are reversed. Differences depending on gender equality norm measures were small but systematically in favour of those capturing the second half of the gender revolution.

## Introduction

A significant trend in industrial countries over the last decades has been distinctly declining fertility rates. This trend has impacted many parts of society, not least in relation to the ageing of societies (Esping-Andersen, 2000). When fewer children are born, an imbalance emerges between the working age population and the elderly population, putting pressure on pension and tax systems. In some societies, the decline in fertility has been particularly pronounced, resulting in fertility rates at ultra-low levels and even more severe consequences of population ageing.

Within the framework of the second demographic transition (SDT), central explanations behind the declining fertility levels involve, on the one hand, structural factors such as women’s increased integration into the labour market, creating new opportunity structures, and, on the other hand, a broader value change stemming from increased economic prosperity, from emphasising traditional values associated with material security, physical safety, and religion towards individualisation, self-realisation, and secular values, all of which imply a weakening of the nuclear family ideal (Lesthaeghe, 1994; Frejka & Westoff, 2008).

However, after decades of historically low fertility levels in many European and other Western countries, research has documented several indications suggesting a weakening of this trend (Esping-Andersen & Billari, 2015). Consequently, scholars have reconsidered the implications of women’s increased employment and highlighted the role of gender equality as a possible factor associated with fertility. Within the theory of the gender revolution, while acknowledging that the transition of women/mothers into employment initially strained the nuclear family and fertility in various ways – a change that placed a heavier burden on employed women as no corresponding change in men’s family behaviour was observed (Goldscheider et al., 2015; Lappegård et al., 2021) – the main message is that this shift should rather be understood as the first half of the gender revolution: the entrance of women into a previously typically male sphere of society. The second half of the gender revolution involves a further transformation of traditional gender patterns: a fundamental change in men’s behaviour as they become increasingly involved in the family and in the care of children. Such a change is presumed to have family-friendly outcomes and hence positive effects on fertility (Goldscheider et al., 2015; Esping-Andersen, 2000).

By using survey data from the International Social Survey Programme’s (ISSP) 2012 module, covering 26 societies in Europe and North America representing different family and gender-equality models, we align to the question of whether and how the progress of gender equality, and specifically the second half of the gender revolution, is associated with fertility ideals.

Compared to the substantial body of work examining actual fertility and fertility intentions, fewer studies have focused on individuals’ fertility ideals and how these may vary across different societal contexts (but see Paul & Grönlund, 2024). Fertility ideals capture an impersonal socially desirable level of fertility rather than an individual’s preferences regarding their own childbearing. Both actual fertility and fertility intentions are more strongly conditioned by individual constraints and short-term societal trends or crises. These measures also exclude individuals who are not yet of childbearing age or who are no longer in the childbearing years. While fertility ideals are not well suited to strictly predicting fertility levels, they may be seen as a reflection of norms concerning a “desirable” societal family size (Philipov & Bernardi, 2011; Sobotka & Beaujouan, 2014; Trent, 1980) and have remained relatively stable over recent decades (Behrman, 2024; Sobotka & Beaujouan, 2014).

Studying fertility ideals enables the inclusion of the entire adult population in the analysis and thus provides an opportunity to compare potential differences among key social groups identified in previous research based on gender, generation, education, and religiosity. In studies focusing on the first half of the gender revolution, results indicated that women, younger generations, the highly educated, and the non-religious were comparatively less child-centred. As described below, these differences were theoretically linked to varying socialisation experiences and opportunity costs across social groups. Applied to a cross-national perspective, this suggests that these group differences exist in a prototypical traditional society in which men are breadwinners and women are responsible for the home and children. However, in a society travelling along the path of the second half of the gender revolution, these differences are expected to narrow due to the expansion of supportive (buffering) policies and gender equality in both the private and public spheres.

The 2012 data do not capture changes that may have occurred in the last years, and recently there appears to be an additional, or new, decline in fertility behaviour (Eurostat, 2026; Begall & Hiekel, 2025). However, previous research suggests that fertility ideals are more stable over time than fertility behaviour, as they are deeply embedded in cultural norms and value systems (Behrman, 2024; Sobotka & Beaujouan, 2014). We therefore believe that data from 2012 are still informative and relevant.^1^

## Gender Equality and Fertility

### Theoretical Perspectives

The relationship between gender equality and fertility has been examined from different theoretical perspectives that, to a significant extent, are cumulative and interconnected, each building upon and refining the insights of earlier approaches. In the following, we present the perspectives relevant to the present analysis. Taken together, they help us understand cross-country variation in fertility ideals, differences among social groups within countries, and how societal-level factors may modify such differences.

One of the most dominating explanations behind the trend of declining fertility is the “second demographic transition” (SDT), a theoretical framework rooted in the writings of Lesthaeghe and Van de Kaa (1986) (Van de Kaa, 1987; Lesthaeghe, 1994). SDT is principally based on two components. The first, building on the modernisation theory developed by Inglehart (1977, 2018), relates to a slowly emerging fundamental ideational shift towards more individualistic values propelled by economic prosperity, affecting preferences regarding family formation and fertility behaviour. A generally increased sense of material and economic security among individuals in modern societies contributes to a stronger focus on self-realisation through educational attainment and career development, which precedes parenthood (Lesthaeghe, 2010). A weaker attachment to traditional family forms paves the way for “new” ones such as cohabitation instead of marriage, and postponement of family formation. This development is expected to be especially pronounced among younger cohorts whose formative years have taken place in societal conditions that emphasise individualistic values (Inglehart et al., 2017), as well as among the highly educated who have effectively focused on self-realisation and invested in higher education. Another factor strongly related to modernisation theory and SDT is secularisation (Lesthaeghe, 1994). Religion has been linked with traditional family values and patriarchal family behaviours (Goldscheider et al. 2014b) and there is strong empirical evidence demonstrating that religious individuals have or intend to have more children than non-religious individuals (Frejka & Westoff, 2008; Buber-Ennser & Berghammer, 2021 and works cited therein). The second component, which appears to have lost influence over time within the SDT framework (Zaidi & Morgan, 2017; Lesthaeghe, 2014), consists of economically oriented perspectives suggesting that fertility levels are expected to decline due to increased opportunity costs of childbearing and associated changes in individual preferences. These opportunity costs are primarily connected to women’s increased investments in higher education and labour force participation.

Social role theory, which centres on the ongoing transformation of gender roles and relations (see e.g., Esping-Andersen & Billari, 2015), shares the basic components of the SDT framework, namely economic opportunity costs and value-based preferences, but it integrates and emphasises the mutual associations between the components. Declining fertility is promoted by a discrepancy between women’s ambitions, interests, and often economically necessary behaviour on the one hand, and traditional social gender roles on the other (McDonald, 2000; Eagly et al., 2000). The social expectation that women should assume responsibility for care and housework while also participating in paid employment implies a dual burden. Importantly, the size of this discrepancy is likely to be more accentuated in conservative countries, where men traditionally participate little in care and housework and institutional support in the form of childcare and elderly care is rudimentary. Put simply, women, especially the highly educated, in conservative countries need to make a choice between having children and pursuing a career (De Laat & Sevilla-Sanz, 2011). Women’s fertility preferences are considered more important than men’s preferences, as the contraceptive revolution of the 1960 s largely shifted fertility control from men to women (Hakim, 2003).

As shown above, the extent of the discrepancy between social expectations, interests, and structural opportunities or barriers is likely to vary across countries. But what societal-level factors are conducive to reducing this discrepancy? Institutional theory suggests that the consequences of a shift towards individualistic values, the nature of increased opportunity costs and changes in women’s social roles, or more broadly gender relations, are contingent on societies’ institutional arrangements (Daly & Rake, 2003; Öun, 2012). In short, through the redistribution of resources and as a carrier of norms (Svallfors, 2007; Bergqvist & Saxonberg, 2017; Lohmann & Zagel, 2016), social policy makes up a framework that defines and influences the roles and relations of women and men and can contribute to alleviating incompatibilities between childbearing and employment (Esping-Andersen, 1999; Esping-Andersen & Billari, 2015). In essence, current and future fertility patterns are expected to be more vulnerable to decline in traditional conservative countries than in countries characterised by widespread gender-egalitarian norms and the adoption of dual-earner family policies. Both factors enhance opportunities to reconcile parenthood and employment, particularly for mothers (Neyer, 2013).

A further advancement of the above reasoning is expressed within the theory of the gender revolution (Goldscheider et al., 2015). The novelty of this approach is that the understanding of gender equality and its implications for fertility goes beyond the role of institutions, which mainly affects the situation for working mothers, and brings in the role of men in the private sphere. In ideal-typical terms, the second half of the gender revolution suggests that a form of gender equality that is particularly family-friendly and supportive of childbearing is achieved when women and men share equal responsibility for housework and childcare, and that both contribute economically to the household. In line with this reasoning, some researchers have argued that the association between gender equality and fertility is expected to be U-shaped rather than linear (McDonald, 2000).

### Previous Research

Previous empirical research on the relationship between gender equality and fertility covers both factors at the individual level and dynamics within couples, as well as factors at the macro level. At the individual level, gender equality has typically been operationalised in terms of the division of housework (Okun & Raz-Yurovich, 2019) or gender role attitudes among individuals (Arpino et al., 2015). At the societal level, gender equality has to a large degree been conceptualised as women’s empowerment in the public sphere, or as the role of the state in reducing work-family conflict (for mothers) (Baizan et al., 2016). In the literature, there is no strong consensus about how gender equality is empirically associated with fertility, but, focusing on processes pertaining to women’s increased employment, the overall tendency is that gender equality measured from this perspective seems to be negatively associated with fertility rather than the opposite, confirming the expectations of the SDT framework **(**Behrman & Gonalons-Pons, 2020).

Regarding research on the individual level, studies show that more gender equal attitudes seem to be associated with fewer children. Jang et al. (2017) conclude that individuals holding traditional attitudes intend to have more children, especially in countries with a higher family expenditure. Still focusing on individual-level fertility intentions, this result is mainly confirmed by Lappegård et al. (2021), but they nuance the measurement of gender equality and show that the relationship depends on whether the attitudinal indicators concern gender relations in the public sphere or mothers’ and father’s roles in the family. Different ways of measuring gender equality and fertility decisions at the individual level may be regarded as responses to stages of change in gender relations (cf. Lappegård et al., 2021). Hence, how gender equality is conceptualised and measured, whether it focuses solely on women’s emancipation, or considers men’s role in the family, influences the relationship with fertility. Puur et al. (2008) conclude that men with egalitarian attitudes regarding men’s family roles tend to have higher levels of desired fertility (but see Westoff & Higgins, 2009). Furthermore, Neyer et al. (2013) show that the association between gender equality and fertility intentions varies depending on which dimension of gender equality is studied. A reversal of the association, i.e., a positive effect of attitudes favouring gender equality on fertility, seems to require high levels of gender equality attitudes overall and a symmetry in attitudes between men and women (Arpino et al., 2015). This argument and empirical evidence are further supported by Goldscheider et al. (2013), focusing on actual fertility in Sweden, who show that an inconsistency between attitudes regarding the division of household work and the actual division of household work reduces the likelihood of continued fertility.

Turning to the influence of societal-level gender equality on fertility, studies have shown that institutions and policies that support gender equality can buffer against a strong negative fertility trend (measured as both actual behaviour and intentions) (Baizan et al., 2016; Mills, 2010). Policies that improve the conditions for parents (mothers) to combine work and childcare increase fertility levels and reduce fertility differences according to the level of education (Baizan et al., 2016). By studying fertility ideals among a large range of countries, also Paul and Grönlund (2024) underline the importance of considering the institutional context. Their findings indicate that societal gender equality can contribute to mitigating the decline in fertility. The same proposition is supported by Frejka et al. (2018). By focusing on the progress of the gender revolution and fertility in 11 countries, they conclude that in countries that have significantly increased gender equality at the societal level by introducing family-friendly policies, cohort fertility declined the least. Also, Kolk (2019) tests the theoretical U-shaped relationship between societal gender equality and fertility but does not find any strong empirical evidence supporting such a claim. However, societal-level gender equality is here conceptualised in terms of women’s political empowerment, without considering the second half of the gender revolution.

Scholars within the gender revolution framework assume that the second half of the revolution has not yet progressed very far across countries. However, they suggest that the emerging contours of increasing male involvement in the traditional female sphere signal the emergence of a significant societal transformation and that such a development would be beneficial for societies both socially and economically (European Commission, 2019; Friedman, 2015; Goldscheider et al., 2015; Gornick & Meyers, 2008). An important aspect of this development is increased fertility, mediated by positive attitudes towards gender equality and the equal sharing of housework and childcare.

Research on men’s involvement in families has expanded over time (Evertsson, 2016; Rush, 2015; Schoppe-Sullivan & Fagan, 2020), and its geographical focus has widened from a rather narrow focus on the USA and the Nordic countries to encompass a broader range of national contexts (Pailhé et al., 2021; Steinbach & Maslauskaitė, 2022; Steinbach & Schulz, 2022; Edlund & Öun, 2023). Over the past decades, parents have increased the time they spend on childcare, irrespective of gender. In general, increases in men’s childcare time have been greater than increases in the time they spend on housework (Pailhé et al., 2021). In a review covering a substantial number of studies on fathers and fatherhood from a wide range of countries worldwide, Seward and Rush (2015) conclude that Scandinavia stands out among Western countries, with Scandinavian fathers assuming greater responsibility for childcare than fathers in other Western countries. A cross-country over-time analysis of predominantly Western countries (*n* = 13), using more recent data, reaches similar conclusions (Sullivan et al., 2018).

## Objectives and Expectations

We will examine the relationship between gender equality and fertility ideals, with a special focus on the role of societal norms regarding men’s participation in care and household work. A norm, or the collective consciousness (Combs & Kripner, 2008), can be understood as the sum of shared preferences expressed by public opinion concerning a specific object or phenomenon (Jary & Jary, 1991). We set out to capture how the prevalence of the second half of the gender revolution, measured through the collective consciousness concerning gender relations, translates into fertility ideals at the societal level as well as among different social groups.

The first objective is to examine the relationship between gender equality and fertility ideals across national contexts. To measure gender equality, we focus on the prevalence of norms embracing the second half of the gender revolution. We expect such gender equality norms to be positively associated with fertility ideals.

The second objective is to compare the analytical value of using direct measures capturing the second half of the gender revolution with that of a “traditional” gender ideology measure focusing on norms related to the consequences of women’s employment for the family. We expect that the measures capturing the second half of the gender revolution will have greater explanatory capacity than the traditional gender ideology measure. The main reason is that contemporary debates across many Western countries are not primarily about whether or not women should be engaged in paid work – most citizens support women’s employment – but the role of men in unpaid work and care and about gender equality in both of these spheres (Frejka et al. 2018; Goldscheider et al. 2014a; Saxonberg 2013).

The third objective addresses differences in fertility ideals across social groups. Previous research focusing on the first half of the gender revolution predicted that actual fertility and/or fertility intentions would be comparatively lower among women, the highly educated, younger people, and the non-religious. When societies enter the second half of the gender revolution, fertility ideals are expected to increase and differences between social groups are expected to diminish, as opportunities for different groups of individuals to realise their fertility ideal become more equal (Testa, 2012).

Concerning gender and educational differences, we expect that differences in opportunity costs of having children become less pronounced and more evenly distributed across these groups. In a similar vein, we expect generational differences to decline, as younger generations are not socialised into the view that fertility is associated with high opportunity costs compared to older generations. Regarding religiosity, we expect religious individuals to report higher fertility ideals than non-religious individuals, although this difference may vary depending on the progress of the gender revolution. Religiosity is associated with traditional family values. Gender equality as defined in the first half of the gender revolution can be perceived as family-unfriendly as it emphasises women’s labour market participation. This may reduce fertility ideals among religious people. As societies progress towards the second half of the gender revolution, gender equality is increasingly viewed as family-friendly, as men’s participation in housework and care is supposed to strengthen families (Goldscheider et al. 2014b), which, in turn, is expected to be associated with higher fertility ideals.

In sum, we expect that group differences in fertility ideals related to gender, education, and age should be less pronounced in societies where gender-equality norms are widespread compared to societies characterised by more traditional gender norms. The influence of religiosity is expected to vary less across gender-equality contexts, in the sense that religious individuals are expected to report higher fertility ideals than non-religious individuals regardless of the prevailing gender-equality norms. However, differences between religious and non-religious individuals are expected to be comparatively larger in societies with strong gender-equal norms.

## Measuring Gender Equality

Empirically, gender equality has been measured using indicators from various sources, including survey-based measures of aggregated individual behaviours and attitudes, institutional policy indicators, and register data on societal outcomes, such as the proportion of women in parliament or the gender balance among senior executives. We use attitudinal measures of gender relations that include both women’s and men’s roles in the family and in paid work. When aggregated, these attitudinal instruments constitute normative expressions of gender equality at the societal level.

As our theoretical framework partly builds on institutional theory, some clarification regarding our choice to use gender equality norms rather than other indicators is needed. Within institutional theory, individual attitudes and behaviours are largely seen as products of national institutions and other contextual factors that may forge substantive interests as well as social norms and identities among the public (Rothstein, 1998; Campbell, 2012). According to this approach, gender equality would logically be best measured using indicators of actual policy arrangements in a society. However, policy indicators related to men’s participation in care and household work within the family are limited.

Other scholars place greater emphasis on the interdependence between norms and institutional-political structures. Social norms are more than passive reflections of structural circumstances; they are also forces that can bring about societal change, *tout court* (Inglehart, 2018; Brooks & Manza, 2007; Mettler & Soss, 2004; Manza & Brooks, 2012; Wlezien & Soroka, 2012). This insight is central and forms one of the basic rationales for analysing public attitudes and experiences, as they constitute a potential force for either change or the preservation of existing societal conditions and inequalities.

“Gender ideologies are sets of widely taken-for-granted cultural beliefs about the essential natures and relative worth of men and women” (Chatillon et al., 2018: 217). They reflect an individual’s level of support for a division of paid work and family responsibilities based on the idea of separate spheres (Davis & Greenstein, 2009). A common approach in survey research for measuring societal-level norms regarding gender equality is to aggregate individual attitudes to the country level.

We apply three different indicators of gender equality context (GEC). One is an index that has been used extensively in previous research, although several variations exist (see e.g., Sjöberg, 2004; Crompton et al., 2005). This index is based on items developed in the 1980 s, mirroring the societal developments documented by scholars concentrating on the effects of the first half of the gender revolution (ISSP, 1988). The index focuses on the perceived consequences, good or bad, for families and children of women’s/wives’/mothers’ participation in paid employment. The role of the man/husband/father is treated as non-variable, as he is implicitly assumed to be a full-time breadwinner. In the empirical analyses, we refer to this indicator as the *Gender ideology index* (GIX).

The other two indicators are derived from the theory of the second half of the gender revolution, in the sense that they extend traditional gender-equality measures, such as the GIX, by including the role of men in the private sphere, particularly in relation to housework and childcare. Designed for the ISSP, 2012 survey, variants of these measures have been applied in previous research (Edlund & Öun, 2016, 2023; Salin et al., 2018). One indicator focuses on a social policy widely regarded by researchers as key to reducing gender inequality by enabling fathers to take greater responsibility for children: parental leave (Gornick & Meyers, 2008; Korpi et al., 2013). Gender equality is measured by the extent to which the parental-leave period should be shared equally between the mother and the father. This measure is denoted *Parental leave equality indicator* (PLEX).

The last indicator builds on theoretical arguments and the scattered qualitative and quantitative empirical evidence supporting them, as suggested by Seward and Rush (2015), Rush (2015), and Goldscheider et al. (2015), and concerns how a prototypical couple with a young child should organise their family and work life. Respondents are asked how the parents should divide the responsibilities of economic provision and unpaid housework and childcare between them. The measure ranges from a traditional division in which the father is the sole breadwinner and the mother assumes full responsibility for the home and childcare, to a progressive family model ideal in which the father and the mother equally share the responsibility for childcare and household work as well as for paid work. This indicator is named *Work/care equality index* (WCEX).

## Data, Variables, Measurements, and Methods

The data come from the ISSP, 2012 module *Family and Changing Gender Roles IV*. The study covers twenty-six societies in Europe and North America; details are provided in Appendix Table A3. All samples are nationally representative of the population aged 18–80 years. This article does not provide sufficient space for a comprehensive description of the data for each country. Information on response rates and sample characteristics for the subsamples is available through GESIS, the official ISSP archive.^2^ However, we wish to emphasise that each country sample meets the quality standards established by GESIS and the methodology committee of the ISSP.

Table 1 presents the characteristics of the variables used in the analyses. Additional descriptive statistics by country are provided in Appendix Table A3. The dependent variable, [Y] *Fertility ideal*, is based on a question asking the respondent: “All in all, what do you think is the ideal number of children for a family to have?” The response alternatives are open; the respondent writes in the preferred number in a box. Extreme values, > 10 children, representing less than 0.1% of the sample were set as non-valid. As shown in the table, the ideal number of children ranks in the following order of popularity: 2, 3, 4, 1, 5, 0, where two or three children are the overwhelmingly most common alternatives. Those preferring five or more children constitute a very small minority. The non-response rate was 7.2%.

**Table 1:** Characteristics of variables

		Percent	(*n*)	Mean	Min	Max	Std Dev	Scale points
*Dependent variable*								
[Y] Fertility ideal				2,39	0	10	0,75	11
	0 no children	0,4	121					
	1 one child	3,9	1045					
	2	60,0	16,176					
	3	29,2	7869					
	4	5,5	1480					
	5	0,7	184					
	6	0,2	58					
	7	0,0	13					
	8	0,0	5					
	9	0,0	7					
	10 ten children	0,0	11					
	Missing value	7,2	2085					
*Individual-level variables*								
[A] Gender	Women	54,6	14,718					
	Men	45,4	12,251					
[B] Education	Q1 high	24,0	6479					
	Q2	26,3	7080					
	Q3	26,7	7202					
	Q4 low	23,0	6208					
[C] Age	18–24 years	9,0	2438					
	25–39 years	23,6	6376					
	40–64 years	48,1	12,960					
	65–80 years	19,3	5195					
[D] Religiosity	0–1 (low to high)			0,31	0	1	0,30	8
*Country-level variables*								
[X1] GIX	0–1 (low to high)			0,59	0,45	0,76	0,08	26
[X2] PLEX	0–1 (low to high)			0,52	0,18	0,85	0,19	26
[X3] WCEX	0–1 (low to high)			0,39	0,06	0,76	0,23	20
(n) valid		26,969		26,969				

When aggregated at the country level, this fertility ideal variable provides a reflection of societal norms regarding fertility, reducing the role of the respondent’s own circumstances, intentions, experiences, and actual fertility (cf. Sobotka & Beaujouan, 2014; Philipov & Bernardi, 2011; Trent, 1980). Studying fertility ideals instead of actual fertility behaviour or intention provides a possibility to include the entire adult population in the analysis, rather than only individuals with children or those of reproductive age.^3^

Two methodological advantages of this approach are, firstly, that it allows for an analysis of how fertility ideals differ across social groups, which may indicate the societal circumstances under which different social groups are more or less likely to specify a higher ideal number of children. Secondly, focusing on fertility ideals provides an opportunity to study individuals who are not yet, or are no longer, of childbearing age at the time of the study. Together with our cross-national perspective, this may provide important insights into societies’ future fertility rates. Examining fertility ideals across countries (see Appendix Table A3), we find that the lowest fertility ideal (< 2.20) are reported in several former socialist Eastern Bloc countries: Czechia, Hungary, Germany-east, and Bulgaria. The highest fertility ideal (> 2.50) is reported in Finland, USA, Norway, Poland, Canada, Iceland, and Ireland – a mix of Nordic, North American and traditionally strong Catholic European countries.

Next, the individual-level variables. *Gender* [A] is a dichotomy. *Education* [B] is measured by completed number of years in education, and the variable is recoded into quartiles, ranging from Q1 (high) to Q4 (low). *Age* is operationalised as age groups [C] and divided into four categories: 18–24, 25–39, 40–64, 65–80, considering two different age groups where family formation usually is planned or occurs. While some theoretical arguments refer to differences across generations, it is not possible to distinguish between age and generation in a cross-sectiocomment these analyses in the text. The results are availnal study. *Religiosity* [D] is based on the following question: “Apart from such special occasions as weddings, funerals, etc., how often do you attend religious services?” The eight-point scale varies from “several times a week or more often” to “never”. Although this variable is on the ordinal level, we use it as ratio-level variable, transformed to vary between 0 (non-religious) to 1 (strongly religious).

The three country-level GEC variables are based on items from the 2012 survey. Mean values for each of these broken down by country are shown in Appendix Table A3. The first, focusing on the first half of the gender revolution, is the [X1] *Gender ideology index* (GIX). It is an additive index (Cronbach’s alpha = 0.78) consisting of six items: (1) “A working mother can establish just as warm and secure a relationship with her children as a mother who does not work”; (2) “A pre-school child is likely to suffer if his or her mother works”; (3) “All in all, family life suffers when the woman has a full-time job”; (4) “A job is all right, but what most women really want is a home and children”; (5) “Being a housewife is just as fulfilling as working for pay”; (6) “A man’s job is to earn money; a woman’s job is to look after the home and family”. The 5-point response scale runs from “strongly agree” to “strongly disagree”. The GIX variable (item 1 reversed) has been transformed to vary between 0 and 1, where lower (higher) values indicate a traditional (progressive) gender ideology. The GIX variable values are aggregated country means. Table 1 indicates that these means vary between 0.45 (Latvia) and 0.76 (Germany-east), with an overall mean of 0.59. The following countries are close to this mean: Belgium, Croatia, Ireland, Slovenia, and the UK.

The next two country-level variables are intended to capture the second half of the gender revolution. The [X2] *Parental leave equality indicator* (PLEX) is constructed from an item formulated as: “Consider a couple who both work full-time and now have a new-born child. If both are in a similar work situation and are eligible for paid leave, how should this paid leave period be divided between the mother and the father?” Response categories: (1) The mother should take the entire paid leave period and the father should not take any paid leave; (2) The mother should take most of the paid leave period and the father should take some of it; (3) The mother and the father should each take half of the paid leave period; (4) The father should take most of the paid leave period and the mother should take some of it; (5) The father should take the entire paid leave period and the mother should not take any paid leave.

In the sample, less than 0.5% of the respondents opted for categories 4 and 5, meaning that when a gender-unequal distribution is preferred, it is the mother that should take all or most of the paid leave. In constructing the PLEX variable, running from 0 to 1, the item was recoded as: 3 = 1 (equal sharing of the parental leave period); 2, 4 = 0.5 (in empirical terms: the mother takes most of the parental leave period); 1, 5 = 0 (in empirical terms: the mother takes the entire parental leave period). The PLEX variable values are aggregated country means. As shown in Table 1, the empirical country means vary between 0.18 (Slovakia) and 0.85 (Sweden), with a mean of 0.52, where the following countries are close to this mean: Poland, Portugal, UK, Canada, and USA.

The final country-level variable [X3] *Work/care equality index* (WCEX) is based on two items. The first asks: “Consider a family with a child under school age. What, in your opinion, is *the best way* for them to organise their family and work life?” Again, the response scale is gender symmetrical and is as follows: (1) The mother stays at home and the father works full-time; (2) The mother works part-time and the father works full-time; (3) Both the mother and the father work full-time; (4) Both the mother and the father work part-time; (5) The father works part-time and the mother works full-time; (6) The father stays at home and the mother works full-time. The second item uses the same response scale but asks which of the options that would be the *least desirable*. As indicated by the answer categories, the alternatives 3, 4 are clearly the most gender equal, and 1, 6 are the most gender unequal. By applying Latent Class Analysis (Magidson & Vermunt, 2001; McCutcheon, 1987) in earlier work (Edlund & Öun, 2023) we distinguished different types of preferred solutions, ranging from very traditional divisions where the father is the single breadwinner and the mother takes full responsibility for the home and care of the child, to progressive family model ideals where fathers and mothers share the responsibility for family and care as well as for paid work. No model could be identified where the father should take a stronger responsibility for the home and children than the mother. Furthermore, the different models that we distinguished can be arranged along a continuous scale ranging from 0 – a traditional gender division: the role of a father as a breadwinner and the role of the mother as a housewife taking care of the child, to 1 – where the roles of fathers and mothers are similar: both contribute to the household equally in both spheres.^4^ Table 1 shows the empirical minimum and maximum values, ranging from 0.06 (Czechia) to 0.76 (Sweden), with a mean at 0.39, where the following societies are close to the mean: Canada, Ireland, and Germany-west.

The empirical analysis is conducted using multilevel analysis, a statistical method that is suitable when exploring relationships between variables that belong to different hierarchical levels – in our case the country-level and the individual level (Hox, 2002; Steenbergen & Jones, 2002).

## Empirical Findings

The results are based on multilevel analyses in which our three measures of gender equality norms (GEC country-level variables) and four individual-level variables are regressed on fertility ideal. Our main interests in the analyses are: (i) the associations between the GEC country-level variables and fertility ideal, (ii) the interactions between the GEC country-level variables and each of the individual-level variables gender, education, age, and religiosity on fertility ideal, and (iii) the extent to which the associations under (i) and (ii) differ across the three GEC variables.

Table 2, Model 1, presents the association between the fertility ideal and the parental leave equality indicator (PLEX).^5^ The coefficient is statistically significant and positive, indicating that individuals living in countries characterised by a strong gender equality norm on average prefer a higher number of children compared to individuals living in countries where the gender equality norm is weak.

**Table 2:** Fertility ideal by parental leave equality indicator (PLEX) (country level), gender, education, age, and religiosity (individual level)

		M1	M2	M3a	M3b	M3c	M3d
Intercept		**2.18**	**2.04**	**2.07**	**2.16**	**2.14**	**2.10**
*Country-level*							
[X2] PLEX	0–1 (low to high)	**0.44**	**0.53**	**0.47**	0.31	0.35	**0.40**
*Individual-level*							
[A] Gender	Women		−0.01	**−0.07**	−0.01	−0.01	0.00
	Men		0	0	0	0	0
[B] Education	Q1 high		−0.01	−0.01	**−0.21**	−0.01	−0.01
	Q2		**−0.08**	**−0.08**	**−0.22**	**−0.08**	**−0.08**
	Q3		**−0.06**	**−0.06**	**−0.19**	**−0.06**	**−0.06**
	Q4 low		0	0	0	0	0
[C] Age	18–24 years		0.01	0.01	0.00	**−0.33**	0.01
	25–39 years		**−0.05**	**−0.05**	**−0.05**	**−0.17**	**−0.05**
	40–64 years		−0.02	−0.02	−0.02	**−0.10**	−0.02
	65–80 years		0	0	0	0	0
[D] Religiosity	0–1 (low to high)		**0.49**	**0.49**	**0.49**	**0.48**	**0.27**
*Interaction-terms*				X2*A	X2*B	X2*C	X2*D
				**0.11**	**0.36**	**0.64**	**0.46**
				0	**0.26**	**0.21**	
					**0.23**	0.16	
					0	0	

Cell entries in **bold** are significant *p* < 0.05.

Individuals (n) = 26,969; societies (n) = 26.

In models M3a-d: for each cross-level interaction, random intercept for each specific individual level variable is included.

Model 2 adds individual-level variables, which allow us to control for sample compositional effects. The results indicate that the effect of the PLEX variable on fertility ideals does not change substantially, suggesting that the results observed in Model 1 were not driven by compositional differences across the country samples. Examining the effects of the individual-level variables on fertility ideals, Model 2 assumes that these relationships do not differ across national contexts. We find that differences between men and women are close to nil, and so are differences between those with the highest and those with the lowest levels of education. Those with intermediate levels of education, however, prefer a comparatively lower number of children on average. With regard to age, the table shows that differences between age groups are quite small; the only age group that deviates from the others is those aged 25–39, who prefer a comparatively lower fertility ideal. Finally, as predicted, religiosity is positively associated with fertility ideals.

In sum, except for religiosity, whenever pronounced associations between individual-level variables and fertility ideals can be observed, it is difficult to fit them into any existing relevant theoretical framework. This applies particularly to the observed differences between educational groups. However, as the theoretical assumptions guiding our analyses suggest that country context matters, we did not expect Model 2 to yield results that were easily interpretable and understandable. As noted above, the primary purpose of including the individual-level variables was to control for sample composition.

We now turn to the interaction between the individual-level variables and the country-level variables, i.e., we are interested in what happens when the relationships between the individual level variables and fertility ideal are allowed to vary across national contexts. Models 3a-3d include interaction terms between each of the individual-level variables and the PLEX variable. Examining these models, we find that all cross-level interactions are statistically significant, indicating that the associations between the individual-level variables and fertility ideals vary systematically across societies depending on how individuals think about gender equality, in terms of shared parental leave.^6^

To interpret the interaction terms adequately, it is necessary to consider the main effects of the included variables simultaneously, which can be a rather cumbersome exercise.^7^ Our chosen strategy for visualising how the associations between the individual-level variables and fertility ideals vary by national gender equality context (GEC) is to present the results in the form of predicted values.

**Fig. 1:**
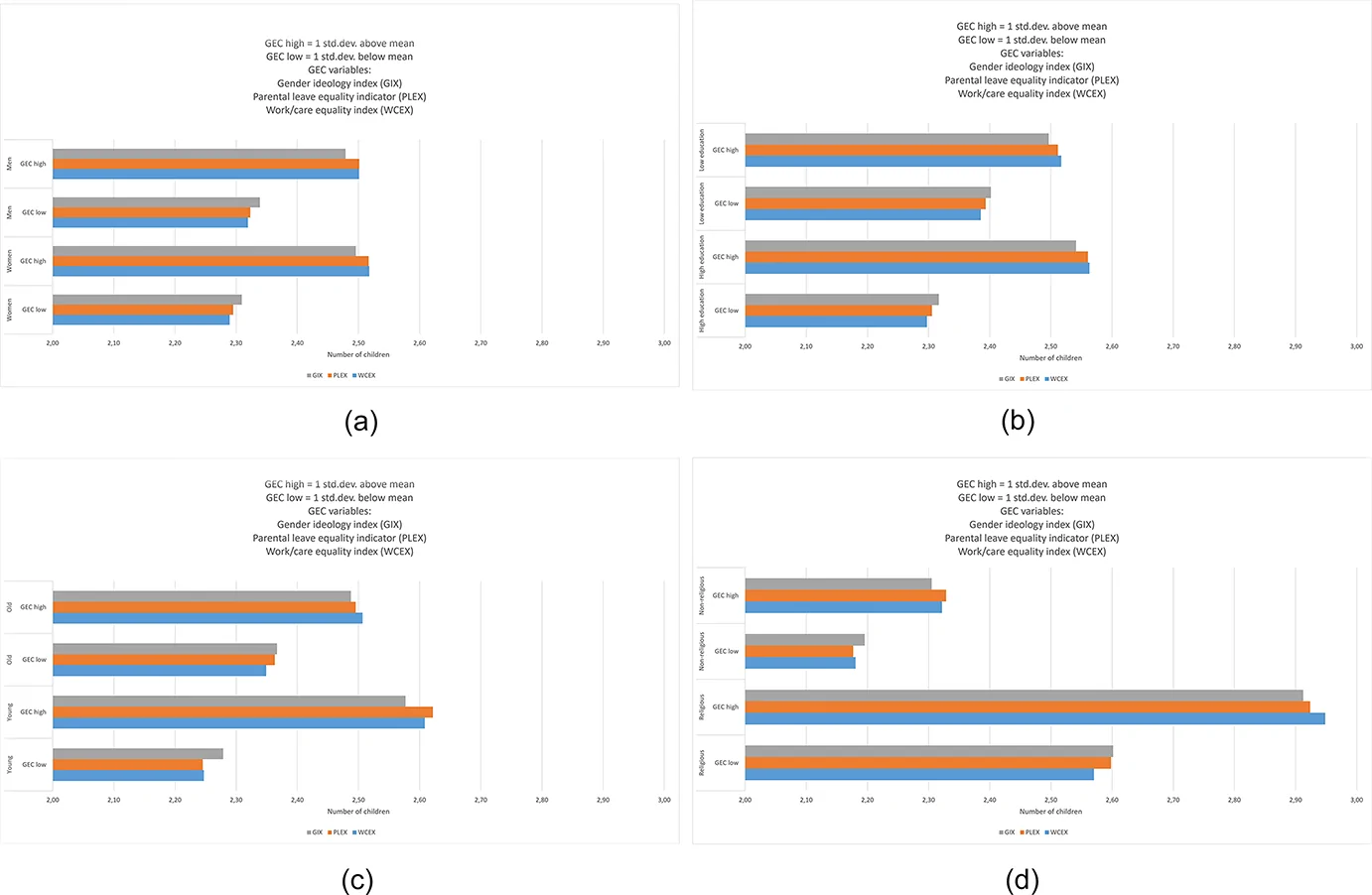
Fertility ideal by gender (a), education (b), age (c), and religiosity (d) by gender equality context (GEC)

Figure 1a–d present the interaction effects on fertility ideal between each of the individual-level variables and the three GEC variables.^8^ Whenever predicted values are mentioned in the text, we refer to those based on the PLEX measure. As contrasting cases (low and high), we use the mean values -/+ 1 standard deviation on the GEC variables, accounting for the observed differences in variation (see Table 1). All individual level-variables, except the one involved in the respective cross-level interaction, are held at their mean values.^9^

Figure 1a shows the relationship between gender and fertility ideals in societies with strong and weak gender equality norms, respectively. Two findings are worth highlighting. First, both men and women in countries with strong gender equality norms report higher fertility ideals than men and women in countries with weak gender equality norms. Second, in gender-equal countries, women’s fertility ideals are slightly higher than men’s, whereas the opposite pattern is observed in gender-unequal countries. The highest fertility ideal is reported by women living in societies characterised by strong gender equality norms, while the lowest are reported by women living in societies where gender equality norms are weak. In sum, these findings suggest that gender equality norms have a substantial impact on fertility ideals. The mean values of bars one and three (counted from the top in the diagram), representing high gender equality contexts, are approximately 2.5 children, whereas the corresponding mean values for low gender equality contexts (bars two and four) are approximately 2.3 children. Compared with these differences, the gender differences are smaller. Interestingly, however, they display opposite patterns across the two contrasting gender equality contexts.

In Figure 1b, the interaction between education and gender equality norms on fertility ideals is examined. We contrast individuals with the highest (Quartile 1) and the lowest (Quartile 4) levels of education. First, irrespective of educational level, individuals living in more gender-equal countries indicate higher fertility ideals than individuals living in less gender-equal societies. Second, as was the case for gender, the relationship between education and fertility ideals differs between countries with low and high levels of gender equality. The group reporting the highest fertility ideal (slightly above 2.5) consists of highly educated individuals living in gender-equal societies, whereas the group reporting the lowest fertility ideal (approximately 2.3) consists of highly educated individuals living in gender-unequal societies.

Figure 1c examines the extent to which older (65–80 years) and younger (18–24 years) cohorts differ in their fertility ideals across national contexts characterised by stronger and weaker gender equality norms. The first observation is that both younger and older individuals living in more gender equal countries report higher fertility ideals than their counterparts living in less gender-equal countries. Moreover, as was the case for gender and education, the association between age and fertility ideals differs across the two modelled ideal-typical contexts, with younger individuals occupying the extreme positions. The highest observed fertility ideal is reported by younger individuals in gender-equal societies (approximately 2.6), whereas the lowest are reported by younger individuals living in gender-unequal societies (slightly below 2.3).

Regarding the interaction between PLEX and age, it is also informative to examine one of the intermediate age groups, namely individuals in their childbearing years (aged 25–39). As shown in Table 2, the interaction term for this group also differs significantly from the reference category (individuals aged 65–80). Predicted values estimated for these groups reveal the same overall pattern, although the differences are somewhat smaller. Individuals aged 25–39 years living in more gender-equal (less equal) societies show a fertility ideal of 2.5 (2.3).^10^

Figure 1 d shows fertility ideals broken down by religiosity (0 and 1) and gender equality context. In contrast to the patterns observed for gender, education, and age, religiosity appears to have a unidimensional association with fertility ideals. Strongly religious individuals report higher fertility ideals than non-religious individuals, both in countries where gender equality norms are strong and in countries where such norms are weak. However, the fertility ideal reported by strongly religious individuals living in gender-equal societies is striking (slightly above 2.9), both compared with religious individuals living in less gender-equal contexts and with non-religious individuals in both types of contexts. For these other groups, mean fertility ideals range from approximately 2.2 and 2.6.

Finally, a comparison of how the effects of group characteristics on fertility ideals are influenced by the GEC country-level variables (Fig. 1a–d) shows that the discriminatory power is consistently stronger for the two measures designed to capture men’s participation in domestic duties (PLEX and WCEX) than for the traditional gender equality measure (GIX). However, it should be recognised that, although the observed differences are systematic, they are not particularly large.

Taken together, these results support the gender revolution theory, both in terms of the positive effect of GEC variables on fertility ideals and the different patterns of association between fertility ideals and social groups (gender, education, and age) observed in societies with less egalitarian gender norms compared with societies characterised by more egalitarian gender norms. Whether these differences are substantial is, to some extent, in the eye of the beholder.

We wish to emphasise two points. First, although we do not consider the group differences to be particularly large, we find it noteworthy that the differences observed in countries characterised by less egalitarian gender norms are reduced and, in some cases, even reversed in countries characterised by more egalitarian gender norms. Second, the gender revolution theory makes explicit causal claims. Given the cross-sectional nature of our design, it is not possible to address causality directly. Nevertheless, the empirical evidence presented here does not contradict the assumptions of the gender revolution theory. At the same time, it does not provide strong empirical support as we do not examine societal developments over time.

## Conclusions

In this article, we examined the relationship between gender equality and fertility ideals, focusing on societal norms about men’s participation in care and household work. We attempted to capture how the prevalence of the second half of the gender revolution, measured through the collective consciousness concerning gender relations, translated into fertility ideals on the societal level (objective 1) as well as among different social groups (objective 3). Moreover, we compared the analytical value of using direct measures capturing the second half of the gender revolution with that of a “traditional” gender ideology measure that focuses only on the first half of the gender revolution (objective 2).

Concerning the first objective, we expected a positive correlation across societies between the degree of norms emphasising gender equality and the level of fertility ideals. We anticipated an especially strong effect of gender equality norms embracing not only women’s emancipation but also the involvement of men/fathers in care and housework on equal terms with women/mothers. Such a development is suggested to be beneficial for families as the family becomes a joint project. Our findings were consistent with these expectations, suggesting that gender equality norms are positively related to fertility ideals. Central to our argumentation is the importance of bringing in measures that explicitly captured the second half of the gender revolution: fathers’ role in the family and care. Thus, we expected that the measures of the second half of the gender revolution would have greater explanatory capacity compared to the traditional gender ideology measure (Objective 2). This argument was not strongly supported as all three indicators of gender equality norms had similar effects. However, the results indicate small but systematically anticipated differences between the measure addressing women’s employment and consequences thereof (GIX), and the two other measures aiming to capture aspects of the second half of the gender revolution (PLEX and WCEX). This indicates that measurements of gender equality that capture gender equality where men’s participation in household work and care is included discriminate somewhat better between social groups’ fertility ideal. One possible explanation for the rather small observed fertility ideal differences between the gender equality norms might be that the second half of the revolution is not yet very advanced across countries. Nevertheless, the PLEX and WCEX measures manage to capture a wider spectrum of gender equality norms, as indicated by their larger response variance compared to GIX and may potentially be useful in future research in the field of gender equality. Moreover, in societies that are beginning to embrace gender-equality norms that include men’s roles in the family, respondents are also likely to endorse attitudes associated with the first half of the gender revolution. This may explain why the different measures appear to reflect similar underlying attitudes in these contexts.

The third objective addressed differences in fertility ideals across social groups. In line with theory and research covering the first half of the gender revolution, we expected that fertility ideals in weak gender-equality contexts would be comparatively lower among women, the highly educated, younger generations (measured here as age groups), and the non-religious. Considering gender and education, fertility ideals were expected to vary due to differences in the opportunity costs of having children. Concerning generational differences, the main mechanism introduced was the differing socialisation experiences of younger and older cohorts. As to religiosity, in line with findings from previous research, we expected that religious individuals would have higher fertility ideals than non-religious individuals. In societies with weak gender equality norms, our observations regarding group differences were broadly consistent with theories about the consequences of the first half of the gender revolution. In these societies, women, the highly educated, the young, and the non-religious on average preferred a smaller number of children than men, the less educated, older individuals, and the strongly religious individuals.

Thereafter, we analysed the extent to which gender-equality norms had the capacity to modify these associations towards less pronounced relationships. Indeed, in societies where gender-equality norms are strong, the empirical findings support arguments advanced by scholars within the second half of the gender revolution framework. The same key groups, namely women, the highly educated, and the young, display comparatively higher fertility ideals, exceeding those expressed by men, the less educated, and older individuals. In general, most groups seem to report higher fertility ideals in countries that have progressed towards the second half of the gender revolution. This can probably be attributed to reduced opportunity costs of having children, especially among women and the highly educated individuals, who are otherwise more exposed to such constraints, a situation that may also reduce generational differences over time, as new generations are not socialised into a reality where fertility is associated with high opportunity costs.

The divide between non-religious and strongly religious individuals stands firm, but it is worth noting that the strongly religious individuals living in societies marked by strong gender-equality norms prefer substantially higher fertility ideals compared to any other social group examined in this study. This result seems to be in line with the findings of Goldscheider’s et al. (2014b) suggesting that the role of religiosity may change depending on the progress of the gender revolution. In societies situated in the first half of the gender revolution, gender equality is perceived as family-unfriendly because it emphasises women’s labour market participation, whereas in societies progressing towards the second half, gender equality is viewed as more family-friendly, as men’s participation in housework and care is assumed to strengthen families.

Notwithstanding our theoretical foundation in institutional literature, we study gender equality without including any institutional indicators. Gender equality would ideally be measured using indicators of actual policy arrangements within a society, but policy indicators related to men’s participation in care and household work within the family are limited. Instead, we emphasise the value of measuring gender-equality norms through the lens of a society’s collective consciousness regarding gender relations, viewing aggregated attitudinal instruments as normative expressions of gender ideology at the societal level. However, our cross-sectional design limits our ability to observe how changes in gender norms correspond to changes in fertility ideals. In relation to the observed group differences, this limitation pertains particularly to our analysis of generations, which had to be operationalised as age groups. The theory of the two-part gender revolution predicts a reversal of fertility decline at the stage of the second half of the gender revolution. With the cross-sectional data at hand, we cannot measure change in the strict sense, but our results indicate that gender-equality norms are associated with higher fertility ideals and less pronounced differences between social groups.

Another limitation is that our data do not capture changes that may have occurred since 2012, a period characterised by a pandemic, growing concerns about climate change, and heightened global political tensions and armed conflicts, conditions that have affected citizens across the countries included in the study. Periods of crises tend to undermine confidence in the future and have historically affected young people’s short-term desire to have children, typically followed by a recuperation in subsequent years (Aassave et al., 2020). Future empirical research may reveal whether recent developments will lead to a sustained decline in fertility or whether total fertility rates will recover over time.

Over the last decade, fertility rates appear to have declined even in Northern European countries, which raises questions about the relationship between gender equality and fertility as proposed by the gender revolution framework (Begall & Hiekel, 2025). However, prior research suggests that fertility ideals are more resistant to change than fertility behaviour, as they are deeply embedded in cultural norms and value systems (Behrman, 2024; Sobotka & Beaujouan, 2014). Nevertheless, sustained declines in fertility may, if persistent over the longer term, be associated with shifting ideals – a question for future empirical research to address. In addition, it is important to note that the second half of the gender revolution remains an ideal-typical model of gender equality, one that even the most gender-equal societies have only begun to approach.

Despite these limitations, if we believe that norms are important for individual behaviour, our study of the association between the prevalence of the second half of the gender revolution and fertility ideals may help us better understand fertility behaviour in different societies. Overall, the results suggest that a shift in gender-equality norms, from weak to strong, within a society potentially has the power to increase overall fertility ideals in the population, as well as the capacity to substantially increase fertility ideals among groups that have previously been identified as “risk groups”: women, the highly educated, and the young.

## Data Availability

All data used in this article are available for the research community for free. See the reference list for details about sources.

## Appendix

See Tables A1 and A2, and A3.

**Table A1:** Fertility ideal by gender ideology index (GIX) (country level), gender, education, age, and religiosity (individual level)

		M1	M2	M3a	M3b	M3c	M3d
Intercept		1.95	1.70	1.79	1.97	1.87	1.90
*Country-level*							
[X1] GIX	0–1 (low to high)	0.77	**1.03**	**0.88**	0.59	0.75	0.68
*Individual-level*							
[A] Gender	Women		−0.01	**−0.18**	−0.01	−0.01	0.00
	Men		0	0	0	0	0
[B] Education	Q1 high		−0.01	−0.01	**−0.50**	−0.01	−0.01
	Q2		**−0.08**	**−0.08**	**−0.35**	**−0.08**	**−0.08**
	Q3		**−0.06**	**−0.06**	**−0.34**	**−0.06**	**−0.06**
	Q4 low		0	0	0	0	0
[C] Age	18–24 years		0.01	0.01	0.01	**−0.65**	0.01
	25–39 years		**−0.05**	**−0.05**	**−0.05**	−0.20	**−0.05**
	40–64 years		−0.02	−0.02	−0.02	−0.16	−0.02
	65–80 years		0	0	0	0	0
[D] religiosity	0–1 (low to high)		**0.49**	**0.49**	**0.49**	**0.48**	−0.24
*Interaction-terms*				X1*A	X1*B	X1*C	X1*D
				**0.29**	**0.81**	**1.11**	**1.26**
				0	0.44	0.25	
					**0.46**	0.23	
					0	0	

Cell entries in **bold** are significant *p* < 0.05

Individuals (n) = 26,969; societies (n) = 26

In models M3a-d: for each cross-level interaction, random intercept for each specific individual level variable is included

**Table A2:** Fertility ideal by work/care equality index (WCEX) (country level), gender, education, age, and religiosity (individual level)

		M1	M2	M3a	M3b	M3c	M3d
Intercept		2.27	2.13	2.16	2.21	2.18	2.18
*Country-level*							
[X3] WCEX	0–1 (low to high)	0.35	**0.45**	**0.39**	0.29	**0.34**	**0.31**
*Individual-level*							
[A] Gender	Women		−0.01	**−0.05**	−0.01	−0.01	0.00
	Men		0	0	0	0	0
[B] Education	Q1 high		−0.01	−0.01	**−0.13**	−0.01	−0.01
	Q2		**−0.08**	**−0.08**	**−0.16**	**−0.08**	**−0.08**
	Q3		**−0.06**	**−0.06**	**−0.13**	**−0.06**	**−0.06**
	Q4 low		0	0	0	0	0
[C] Age	18–24 years		0.01	0.01	0.00	**−0.17**	0.01
	25–39 years		**−0.05**	**−0.05**	**−0.05**	**−0.09**	**−0.05**
	40–64 years		−0.02	−0.02	−0.02	−0.06	−0.02
	65–80 years		0	0	0	0	0
[D] religiosity	0–1 (low to high)		**0.49**	**0.49**	**0.49**	**0.48**	**0.31**
*Interaction-terms*				X3*A	X3*B	X3*C	X3*D
				**0.10**	**0.29**	**0.44**	**0.51**
				0	**0.18**	0.08	
					0.15	0.10	
					0	0	

Cell entries in **bold** are significant *p* < 0.05

Individuals (n) = 26,969; societies (n) = 26

In models M3a-d: for each cross-level interaction, random intercept for each specific individual level variable is included

**Table A3:** Fertility Ideal [Y], GIX [X1], PLEX [X2], WCEX [X3], by Country

Country	Fertility ideal	Fertility ideal	GIX	PLEX	WCEX	(*n*)
	Mean	Std. Dev.	Mean	Mean	Mean	
Austria	2.21	0.72	0.53	0.42	0.17	999
Belgium	2.28	0.78	0.60	0.65	0.52	1853
Bulgaria	2.18	0.57	0.45	0.22	0.07	913
Canada	2.64	0.86	0.62	0.54	0.37	383
Croatia	2.49	0.85	0.58	0.38	0.31	928
Czechia	2.04	0.62	0.52	0.20	0.06	1648
Denmark	2.44	0.75	0.75	0.70	0.70	1262
Germany-west	2.29	0.69	0.63	0.68	0.37	1109
Germany-east	2.14	0.57	0.76	0.66	0.67	521
Finland	2.52	0.90	0.66	0.70	0.54	1033
France	2.42	0.66	0.65	0.68	0.52	1962
Hungary	2.13	0.69	0.46	0.21	0.10	890
Iceland	2.86	0.65	0.68	0.72	0.72	870
Ireland	2.95	0.88	0.62	0.56	0.38	970
Latvia	2.41	0.69	0.45	0.27	0.12	921
Lithuania	2.33	0.68	0.48	0.28	0.07	968
Netherlands	2.41	0.79	0.63	0.69	0.65	978
Norway	2.57	0.73	0.69	0.69	0.70	1310
Poland	2.58	0.69	0.51	0.50	0.30	859
Portugal	2.23	0.72	0.55	0.51	0.34	864
Slovakia	2.35	0.71	0.50	0.18	0.07	1056
Slovenia	2.40	0.69	0.60	0.38	0.50	901
Sweden	2.39	0.64	0.71	0.85	0.76	901
Switzerland	2.37	0.67	0.54	0.60	0.50	1061
UK	2.29	0.73	0.59	0.51	0.24	667
USA	2.53	0.88	0.56	0.53	0.26	1142

Detailed variable descriptions are shown in Table 1

## Footnotes

1 Recently, the ISSP released data from 2022. However, in the 2022 dataset, several countries relevant to this study, in particularly those representing the market-oriented family model/liberal welfare state, are missing.

2 www.gesis.org/en/issp/data-and-documentation/family-and-changing-gender-roles/2012.

3 It is worth noting that the fertility ideal across countries recorded in the ISSP, 2012 dataset correspond quite well with observed country differences in total fertility 2012. Pearson’s *r* = 0.56; Spearman’s ρ = 0.52, *n* = 25 (the two Germanies collapsed, weighted average). However, fertility ideals are generally higher than total fertility rates. Although this is a crude measure, the strength of the correlations is reassuring from the perspective that the ranking of countries based on the fertility ideal is associated with the country ranking of the total fertility rate. Source and information on the total fertility measure: OECD (2024).

4 In Edlund and Öun (2023) the latent class analysis revealed six clusters. Three were different versions of a traditional family ideal (C1; C2; C3), where the mother takes care of the home and children and the father is a breadwinner. The fourth cluster (C4) captured those believing that the mother should work part-time while the father should work fulltime. The last two clusters were gender equal. One cluster (C5) consisted of those favouring a solution where both parents work fulltime and the other cluster (C6) captured those preferring that both the mother and the father should work part-time and devote equal time to the household and care. In the article, it was acknowledged that in deciding the key parameters and their weights for creating a continuous measure, there was an element of arbitrariness involved. Therefore, four different measurements were created. However, an analysis on the country-level showed that all measurements correlated strongly (lowest value of *r* = 0.99). In this article, we use a measurement applying the following weights for cluster membership probabilities: [WCEX=C1*0 + C2*0 + C3*0 + C4*0.5 + C5*1 + C6*1]. For a detailed description of the cluster characteristics and measurements, see Edlund and Öun (2023).

5 As the results of the multilevel analyses using the WCEX and GIX variables do not differ extensively from those obtained using the PLEX indicator shown in Table 2, we do not comment these analyses in the text. The results are available in Appendix Tables A1 and A2.

6 Interaction-terms between PLEX and: (a) gender F = 5,709, sig = 0,03; (b) education F = 4,106, sig = 0,01; (c) age F = 10,556, sig < 0,001; (d) religiosity F = 4,420, sig = 0,046.

7 Illustrating with a short example, the significant interaction term Gender*PLEX indicates that the slopes (the ideal fertility rate) for women and men are significantly different when moving from a highly gender unequal-norm society to a highly gender equal-norm society. In concrete terms: in the former type of society, men prefer a higher fertility ideal compared to women. In the latter type of society, the relationships are reversed. Accordingly, when moving closer to the mean of the PLEX variable, gender differences are negligible. The same pattern can be observed for age and education. The main conclusion regarding these interaction variables is that when they are significant, they indicate that relationships between each of the background variables and fertility ideals are largest in both ends of the GEC variables scales. This means that in societies where the GEC variables are close to the mean, differences between social groups are negligible.

8 The results using the WCEX and GIX measures (Tables A1, A2) differ to some extent from the reported PLEX measure in Table 2. For the former measures, the main effect of the context variables is not significant in Model 1. However, controlling for individual level variables (Model 2) the main effect of both WCEX and GIX are significant. For Models 3a-3d, all cross-class interactions are significant irrespective of which gender equality country-level measure applied. Level of significance: *p* < 0.05.

9 We have also estimated predicted values based on the empirical minimum and maximum values. The results obtained from these analyses did not deviate from those reported in Diagrams 1a-1d. To check the robustness of the reported associations in Table 2, A1, and A2, we performed identical analyses except that the country-level variables were recoded into quartiles and used as categorical variables. The results were strikingly similar, indicating that the observed relationships are not driven by single outlier countries.

10 For both the WCEX and the GIX measures, this age group does not significantly deviate from the reference category.
